# An ancient immunity gene duplication in *Daphnia magna*: RNA expression and sequence analysis of two nitric oxide synthase genes

**DOI:** 10.1016/j.dci.2009.04.006

**Published:** 2009-09

**Authors:** Pierrick Labbé, Seanna J. McTaggart, Tom J. Little

**Affiliations:** University of Edinburgh, Institute of Evolutionary Biology, School of Biological Sciences, Ashworth Laboratory, Kings Buildings, Edinburgh EH9 3JT, UK

**Keywords:** Invertebrate immunity, Innate immunity, Nitric oxide synthase, *Pasteuria ramosa*, Pathogen, Parasite

## Abstract

NO (nitric oxide) is a highly reactive free radical gas thought to play a major role in the invertebrate immune response by harming pathogens and limiting their growth. Here we report on studies of nitric oxide synthase (NOS) genes in the crustacean *Daphnia*, one of the few non-insect arthropod models used to study host–pathogen interactions. While the NOS gene is found as a single copy in other invertebrates, we found two copies (NOS1 and NOS2), which a phylogenetic reconstruction showed to be the result of an ancient duplication event. Both genes bear features commonly found in invertebrate NOS, however, the two genes differ in their rate of evolution, intraspecific polymorphism and expression level. We tested whether the more rapid evolution of NOS2 could be due to positive selection, but found the rate of amino-acid substitutions between *Daphnia* species to be compatible with a neutral model. To associate NOS or NO activity with infection, we performed infection experiments with *Daphnia magna* and one of its natural pathogens (the bacterium *Pasteuria ramosa*). In one set of experimental infections, we supplemented *D. magna* with l-arginine, the NOS substrate, or with l-NAME, a NOS antagonist, and found this to result in lower and higher infection levels, respectively, which is at least compatible with the notion that NO may aid defence against *Pasteuria*. A second experiment indicated that NOS transcription does not increase following exposure to *Pasteuria*. Thus, the function of NOS in *Daphnia* immunity remains uncertain, but the pattern of gene duplication and subsequent divergence suggests evolution via neo- or subfunctionalization.

## 1 Introduction

Nitric oxide (NO) is a highly reactive free radical gas produced by the conversion of l-arginine into l-citrulline by nitric oxide synthase (NOS). Most of what is known about the biological role of NO comes from studies of vertebrates, where it has been shown to be involved in neurotransmission, inflammation and host defence (see review in [1]). With respect to host defence, the reactivity of NO with oxygen and oxygen-related reactive intermediates yields numerous species with enzymatic and DNA-damaging properties that make NO toxic to many pathogens [1–3]. Three main forms of NOS have been found in vertebrates: two constitutive forms, neuronal (eNOS or NOS1) and endothelial (eNOS or NOS3), which are expressed in almost all the cells of the body [4], and an inducible form (iNOS or NOS2, see [2,4] for a review). iNOS is expressed during inflammation or infection and increases the quantity of NO by 100–1000 times [2]. However, NO appears to be a “double-edged sword” as at high concentrations it can also damage host tissues [1,2,4,5].

**Fig. 1:**
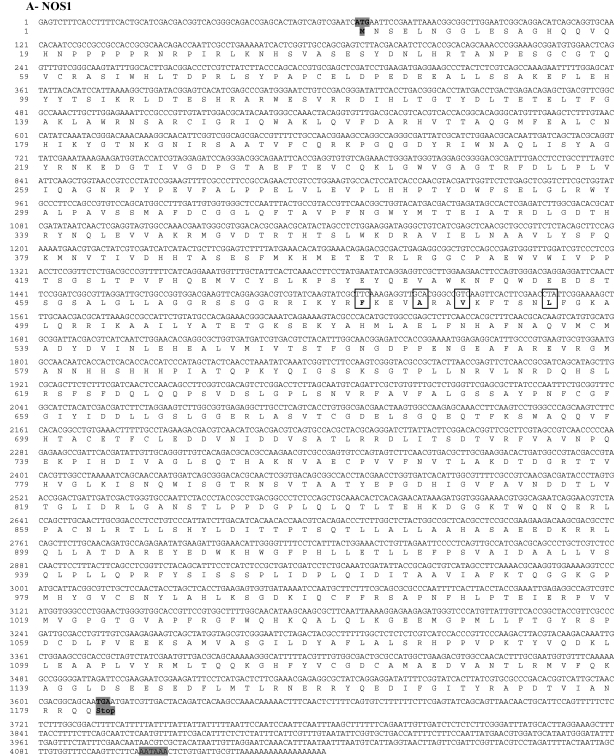

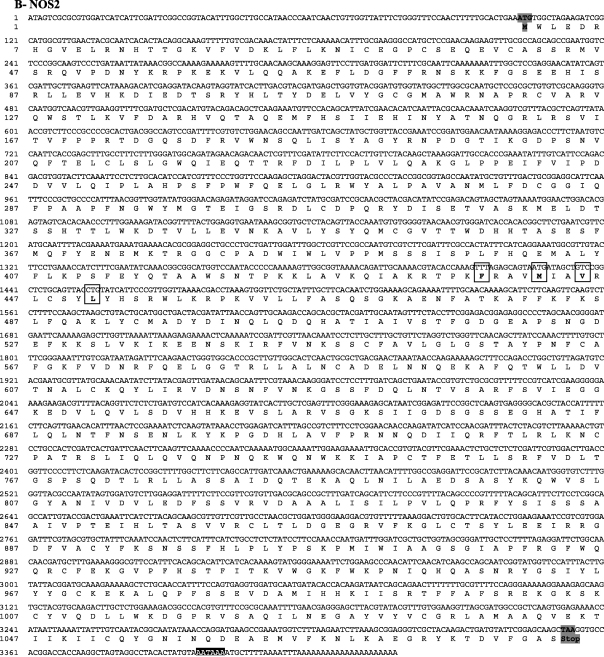
Nucleotide (above) and deduced amino-acid (below) sequence of *D. magna* (A) NOS1 and (B) NOS2. The nucleotide sequences are numbered from the first base at the 5′ end of the transcript. The first methionine (M) is numbered on the first deduced amino-acid of the transcript and the Stop codon is also highlighted (Stop). The hydrophobic residues corresponding to Ca^2+^-dependent clamodulin-binding 1-5-8-14 Type A motif [43] are boxed. The polyadenylation (AATAAA) signal is black highlighted. As expected, no signal peptide was detected using the online software SignalP 3.0 in any of the protein sequences [http://www.cbs.dtu.dk/services/SignalP/, [56]].

Recent years have seen renewed interest in invertebrate immunity (Fig. 1, in [6]), motivated by the importance of invertebrates as vectors or intermediate hosts of human pathogens, and due to their utility as models for understanding coevolutionary processes. Understanding the role of NO in the invertebrate immune system has been part of this surge, but it is presently less well-studied than other effectors such as antimicrobial peptides or prophenoloxidase (proPO). The NOS gene has been sequenced in a range of invertebrates, including insects, molluscs and crustaceans (e.g. [7,8–11]), and in most species, a single NOS gene copy has been detected. However, in the genome of the crustacean *Daphnia pulex* two NOS genes have been described, located on different genomic scaffolds [12].

The role of NO in limiting the impact of pathogens has been demonstrated in a range of invertebrate species. For example, NO helps limit *Plasmodium berghei* development in the mosquito *Anopheles stephensi* [11], it is probably involved in killing *Schistosoma mansoni* in the snail *Biomphalaria glabrata* [13], and it is implied in *Drosophila melanogaster*'s response to parasitoids [14,15]. Additionally, NOS expression has been shown to rise following infection in various organisms [7,8,11,13,16–20]. Our model is the crustacean *Daphnia magna*, a filter-feeding cladoceran that reproduces by cyclical parthenogenesis and is common in freshwater ponds, and which is one of the few non-insect arthropod models used to study host–pathogen interactions. Little is known about the genetic basis of its immune response to pathogens, although phenotypic responses to natural pathogens have been thoroughly investigated. Among the numerous pathogens of *D. magna* (see review in [21]), the gram-positive bacterium *Pasteuria ramosa* is an obligate endopathogen of *D. magna* that is well-studied in both the laboratory and field where it naturally infects *D. magna* (e.g. [22–26]). It permits relatively easy experimentation, and thus it has been shown that resistance to *P. ramosa* depends on host and pathogen genetic background [27], and is mediated by environmental effects [21,28–31].

To better understand the genetic and immunological basis of resistance to pathogens in *D. magna* (see also [26]), here we report on studies of *Daphnia* NOS genes. We took advantage of the recently sequenced genome of a *D. pulex* (http://daphnia.cgb.indiana.edu/), in which two NOS genes have been described [12], to acquire the full cDNA of NOS in *D. magna*. We also found NOS in two copies in this species. We compared deduced NOS amino-acid sequences in *Daphnia* with those of other arthropods to test whether the *Daphnia* genes show signs of functional differentiation, as might be predicted following a gene duplication. As some allelic variation of NOS has been reported to be associated with parasite infection in *A. gambiae* [32], we also analysed polymorphism of *D. magna* NOS cDNAs for 14 genotypes originating from various locations in Europe, eight of which are known to differ in their level of resistance to the pathogen *P. ramosa* [27]. Finally, we quantified the relative expression levels of NOS in *D. magna* and experimentally investigated the potential for NOS/NO to play a direct role in resistance to pathogens.

## 2 Materials and methods

### 2.1 RNA isolation and cDNA sequencing of *D. magna* NOS

Recently, a related species to *D. magna*, *D. pulex*, was subject to full genomic sequencing. To identify conserved regions for designing primers for use in the polymerase chain reaction (PCR) in *D. magna*, we used gene models of the two NOS cDNAs from *D. pulex* [12] and 12 sequences collected from various public libraries: (i) insects: (a) lepidopteran: *Manduca sexta* (GenBank AAC61262), *Bombyx mori* (REFSEQ NP_001036963); (b) dipteran: *D. melanogaster* (GenBank AAF25682), *A. stephensi* (GenBank AAC68577); (c) hymenopteran: *Apis mellifera* (REFSEQ NP_001012980); (d) hemipteran: *Rhodnius prolixus* (GenBank AAB03810), *Acyrthosiphon pisum* (REFSEQ XP_001946209); (e) orthoptera: *Gryllus bimaculatus* (DBJ BAH14964); (f) coleoptera: *Luciola cruciata* (DBJ BAF63161), *Tribolium castaneum* (REFSEQ XP 967195); (ii) crustaceans, decapoda: *Gecarcinus lateralis* (GenBank AAT46681); (iii) chelicerata, acari: *Ixodes scapularis* (GenBank EEC05792). These sequences were also used to draw an interspecific phylogeny of NOS proteins, as described below.

Four 5-day-old *Daphnia* were pooled in 200 μL RNAlater™ (Ambion). RNA was extracted using the RNAeasy midi Kit (Qiagen), according to the manufacturer's instructions and further purified with RNAse-Free DNAse (Promega). Two microliters of RNA (∼200 ng/μl) was reverse-transcribed into cDNA using the Promega Reverse Transcription System kit according to the manufacturer's instructions. cDNA was diluted 5-fold with H_2_O, and its purity assessed [26]. Initial *D. magna* NOS cDNA sequences were acquired using gene-specific degenerate primers, but to obtain the 5′ and 3′ ends of the *D. magna* cDNAs, RACE-PCR was performed with the GeneRacer™ Core kit (Invitrogen). As in *D. pulex*, two homologous sequences were found and named Dmag-NOS1 and Dmag-NOS2. The fragments were assembled using the Geneious Pro 4.0.2 software [33].

The complete *D. magna* cDNA data were used to refine the *D. pulex* NOS1 and NOS2 gene models (Supplementary Materials S1), for use in subsequent analyses. *D. pulex* and *D. magna* NOS protein sequences were aligned to homologous sequences from all other available invertebrate taxa using the ClustalW algorithm within MacVector v7.2.3, and corrected by eye. The completed multiple sequence alignment was used to infer a phylogeny using MrBayes 3.1.2. The mixed model option was used to choose the amino-acid substitution model, a gamma rate distribution parameter estimated from our dataset, and saving every 100th tree. Two parallel Markov chains were run simultaneously in each of two runs. Tree length, amino-acid model, log-likelihood score and alpha value of the gamma distribution were examined in the program Tracer v1.3 prior to the termination of MrBayes to ensure that all parameters had reached stationarity. Saved trees from after the burn-in were summarised and posterior probabilities estimated. The resulting tree was visualized with the program Treeview (v1.6.6, http://taxonomy.zoology.gla.ac.uk/rod/rod.html).

To test the prediction that the *Daphnia* NOS genes are evolving at a different rate compared to other invertebrate NOS genes, we first tested whether rate heterogeneity was present within the tree using the program PAML (version 3.14, [34]). The fit of two evolutionary models was subsequently tested, one that allowed the *Daphnia* NOS clade (containing NOS1 and NOS2) to evolve at a different rate compared to all other clades, and a second that allowed each of NOS1 and NOS2 to evolve at different rates compared to the rest of the tree. In all comparisons, as is standard in PAML analyses, a significant difference between models was tested with a likelihood ratio test.

Next, to evaluate what type of selective pressure NOS genes have experienced, the ratio of non-synonymous substitutions per non-synonymous site to synonymous substitutions per synonymous site (Dn:Ds) was calculated between *Daphnia* species. When Dn:Ds is >1, divergence between species is considered to be due to adaptive evolution. In addition to the full gene length comparison between *D. magna* and *D. pulex*, comparisons of two shorter fragments (549 nt from NOS1 and 660 nt from NOS2) between *D. parvula* (a close relative of *D. pulex*) with each of *D. pulex* and *D. magna* were also included.

In the NOS1 analysis, nucleotides encompassing amino-acids 1163–1346 were analysed. This region includes the NADPH ribose domain and a portion of the NADPH adenine domain as well as two interdomain regions that show low sequence homology among the taxa examined. In the NOS2 analysis, nucleotides encompassing amino-acids 621–841 were analysed. This region includes part of the BH4 domain and the camodulin domain, as well as two interdomains that show low sequence homology among the taxa examined.

### 2.2 Sequence polymorphism in *D. magna* NOS

From the complete *D. magna* NOS cDNA sequences, Primer3 (http://www.bioinformatics.nl/cgi-bin/primer3plus/primer3plus.cgi) was used to design nine or 10 pairs of primers to PCR amplify NOS1 and NOS2, respectively (see sequences in Table 1). All primer pairs are gene-specific and amplify overlapping ∼500–700 bp fragments. Independent PCR were performed for each primer pair (30 cycles, 92 °C for 30 s, 52 °C to 56 °C for 30 s and 72 °C for 1 min) using the Bioline *Taq* DNA polymerase kit. Sequence polymorphism was studied in the same 14 *Daphnia* clones used in Labbé and Little [26]. Briefly, six clones (GG3, GG4, GG7, GG8, GG13, and GG15) were collected in Germany, four clones (KA51, KA5, KA24 and KA47) in Scotland, two clones (BelD1 and BelD3) in Belgium, the last two clones being the reference clones Mullinb3 (Munich, Germany) and Xinb1 (Finland). PCR products were purified and sequenced as in Labbé and Little [29]. All *D. magna* sequences were assembled using the Geneious Pro 4.0.2 software [33]. Intraspecific cDNA alignments were computed using the online software Multalin (http://www.bioinfo.genotoul.fr/multalin/multalin.html, [35]).

### 2.3 NOS expression

RNA was extracted and purified from a mix of 4 crushed *Daphnia* from which cDNA was synthesized as described above (RNA concentration was ∼150 ng/μl). Relative Real-Time Quantitative PCR (RT-QPCR) was implemented using the Roche LightCycler^®^ 480. A ∼100 bp fragment of *actin*, a house-keeping reference gene, was amplified using primers from Heckmann et al. [36]. Note that a second common control gene (*GAPDH*) was also employed but as results did not differ from those obtained with *actin*, only the latter are reported. A 99 bp fragment of Dmag-NOS1 and a 106 bp fragment of Dmag-NOS2 cDNAs were amplified separately using gene-specific primers (NOS1-QF 5′GAGCTCTTCAACCACGCTTT 3′/NOS1-QR 3′AGACGTCACGATCATCACCA 5′ and NOS2-QF 5′AGTCCGATTTTCGTGTCTGG 3′/NOS2-QR 3′ACCTCGGTGAATTGGACATT 5′, respectively). For each NOS gene and each individual, 2 μl of cDNA (1 μL for *actin*) and 0.5 μL of each primer (10 mM) were added to 8 μL of SYBR Green I Master mix (Roche). The quantification of each gene (NOS1, NOS2 and *actin*) was done on separate plates. Cycling conditions were as follows: 95 °C, 5 min followed by 50 cycles of 95 °C for 10 s, 58 °C for 10 s and 72 °C for 10 s. Quantifications of NOS1 and NOS2 relative to *actin* were performed using the Roche LightCycler^®^ 480 software, using the maximum secondary derivative method.

### 2.4 NOS/NO and host resistance to pathogens

#### 2.4.1 NOS expression when challenged by a pathogen

Expression of both NOS genes was further analysed in *D. magna* clone GG4 experimentally exposed to *P. ramosa*. The pathogen spores (Sp1) originated from a single, wild caught infected female and have been used to infect *Daphnia* in the laboratory for over a decade (Sp1, [27]).

Using the conserved cDNAs from the same host individuals from a previous analysis of the proPO gene [26], RT-QPCRs were implemented on each individual with both NOS1 or NOS2 specific primers as described above to compare Dmag-NOS1 and Dmag-NOS2 expression between *Daphnia* exposed or not-exposed to *P. ramosa* at six time-points following exposure. Details of the protocol can be found in Labbé and Little [26]. Briefly, after they spent 3 generations in standard conditions to equilibrate environmental effects, replicates of four 5-day-old *Daphnia* were exposed to a solution of *P. ramosa* spores or to a sham solution (18 replicates per treatment) for 2 h in 1.5 ml eppendorf tubes. After these 2 h, the *Daphnia* were placed in a new jar and reared normally. Three jars for each treatment were randomly collected 1, 2, 6, 12, 24 and 48 h after the end of the exposure treatment. Additionally, three jars were also collected at time 0, prior to exposure, as controls. For each time-point, the four *Daphnia* were transferred to one eppendorf tube with 200 μl of RNAlater™ (Ambion), and stored at −20 °C for later extraction. Three replicates of each treatment were kept in rearing conditions until day 16, to estimate infection success. No supplement with a potential effect on NOS was added during this experiment.

The NOS gene expression data were fitted to the general linear model (GLM): log(Activity) = Exposure + Time + Exposure:Time, where Exposure:Time represents the interaction between Exposure, a categorical variable with two levels (exposed/not-exposed), and Time, a continuous variable (h). Log-transformation of the response variable ensured the normal distribution of residuals. The initial model was simplified according to Crawley [37]. Models were compared using *F*-tests. Analysis was performed using the R package (http://www.r-project.org/).

#### 2.4.2 NOS substrate manipulation

To further assess whether NOS/NO might play a role in resistance to pathogens, *D. magna* were exposed to *P. ramosa* in the presence or absence of l-arginine, the substrate required for NOS activity and for which availability is likely to be limiting [11,20]. The water of the potential hosts was supplemented with l-arginine, our prediction being that increasing availability of l-arginine would limit the establishment of infection.

Six replicates of host clone GG4 were kept under controlled conditions for three generations prior to experimentation. These controlled conditions were 20 °C, a light:dark cycle of 16:8 h, and 5 × 10^6^ cells of chemostat grown algae (*Scenedesmus* sp) per *Daphnia* per day as food. Replicates contained 5 females from the same clutch, in a 200 ml jar of *Daphnia* medium [38]. Each generation was seeded using female newborn from the 3rd or 4th clutches. For the experiment, newborn from each of the six replicate jars were split across twelve treatments. These treatments were: no pathogen spores and *Daphnia* medium adjusted to contain 0, 1, 7.5, 15, 30 or 60 mg/ml of l-arginine (Sigma–Aldrich A8094) or 1 × 10^4^ Sp1 pathogen spores and *Daphnia* medium adjusted to contain the same six levels of l-arginine. The infection period lasted 24 h, during which the *Daphnia* were not fed. The jars used for the infection period were 50 ml in volume. On day 4, each group of five *Daphnia* were transferred to a jar containing 200 ml of *Daphnia* medium (with no l-arginine) and fed 5 × 10^6^ cells of algae per *Daphnia* per day until the end of the experiment at day 30. *Daphnia* were transferred into fresh water three times per week. Infection status of each host was determined by eye and the presence of pathogen spores was later verified by crushing dead hosts and examining them under a microscope at 40× magnification. Results were analysed with general linear models with binomial errors in the R package. The response variable was the proportion of hosts infected. Dose of l-arginine was the categorical explanatory variable.

This experiment was repeated, but 0, 7.5 or 60 mg/ml of l-NAME were added (instead of l-arginine). This compound competes for the l-arginine binding site of NOS and inhibits the reaction, so that the addition of l-NAME is predicted to increase the probability of infection. In this experiment there were eight replicate jars per treatment and per clone and 5 *Daphnia* per jar. Three different clones were used, GG4, GG3 and GG15 (all were sampled in the same pond, [27]). The number of parasite transmission spores added and the other conditions were identical to the previous experiment. Analysis was performed using the R package on the proportion of hosts infected using the GLM with binomial error: proportion infected = Dose + Clone + Dose:Clone, where Dose and Clone are the categorical explanatory variables. The initial model was simplified according to Crawley [37]. Models were compared using *F*-tests.

## 3 Results and discussion

NO may be one of the most general immunity effectors [2], and yet, compared to antimicrobial peptides (AMP) or prophenoloxidase (proPO, [39,40–42]), studies of NO are limited. NOS, the enzyme responsible for NO production, usually occurs as a single copy (e.g. [7–11]), with the exception of *D. pulex* where two gene copies encoding NOS are present [12]. In *D. magna*, we also found two genes encoding NOS proteins (Dmag-NOS1 and Dmag-NOS2). We assembled a total of 4141 bp and 3438 bp of cDNA for Dmag-NOS1 and Dmag-NOS2, respectively. Dmag-NOS1 contains a 67 bp 5′ untranslated region (UTR), a 528 bp 3′ UTR containing the poly-A tail and the polyadenylation signal, and an open-reading frame (ORF) of 3546 bp corresponding to a deduced protein of 1182 amino-acids (MW = 132.02 kDa, p*I* = 6.05, Fig. 1A). Dmag-NOS2 contains a 102 bp 5′ UTR, a short 87 bp 3′ UTR containing the poly-A tail and the polyadenylation signal, and an ORF of 3249 bp corresponding to a deduced protein of 1083 amino-acids (MW = 122.97 kDa, p *I* = 8.67, Fig. 1B). Both Dmag-NOS1 and Dmag-NOS2 possess conserved domains and active sites typical of NOS proteins. In particular the four hydrophobic residues corresponding to the Ca^2+^-dependent calmodulin-binding motif were found in both sequences (Fig. 1A and B; [43]). The sequences of Dmag-NOS1 and Dmag-NOS2 have been deposited in NCBI GenBank under the accession number FJ593039 and FJ593040, respectively.

**Fig. 2:**
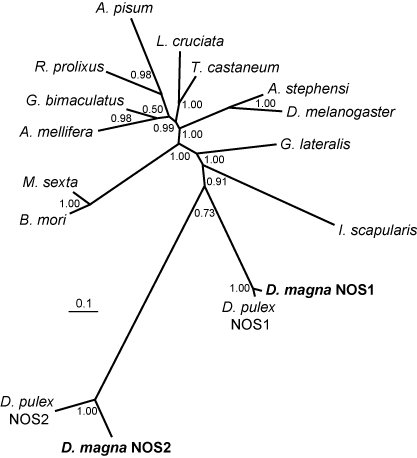
Bayesian phylogeny of the nitric oxide synthase (NOS) gene from available insect and crustacean sequences, numbers at the nodes are posterior probabilities. See text for the reference sequences of the various organisms. Dmag-NOS1 and Dmag-NOS2 are in bold. The scale for 0.1 amino-acid substitutions per site is indicated.

**Fig. 3:**
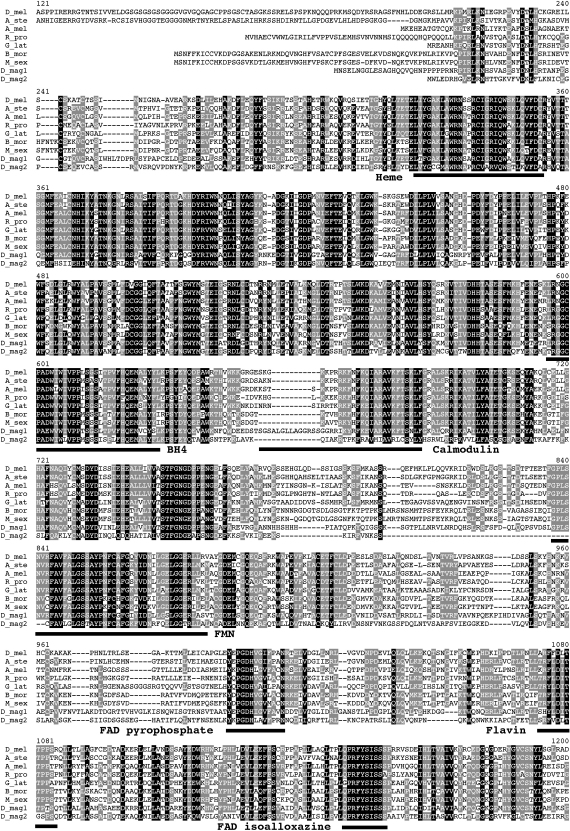

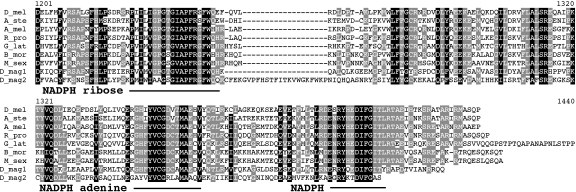
Multiple alignment for conserved motives of several arthropod NOS amino-acid sequences. See text for the reference sequences of the various organisms; for reasons of print space not all the sequences used are presented but only a representative selection of the diversity (in particular as *D. pulex* and *D. magna* are relatively close, the *D. pulex* sequences are not shown). Conserved amino-acid in more than 90% of the sequences are black shaded, those conserved in more than 50% of the sequences are grey shaded. Binding sites to heme, tetragydrobiopterin (BH4), calmodulin, FMN, FAD pyrophosphate, FAD isoalloxazine, flavin, NADPH ribose, NADPH adenine and NADPH are underlined.

The clustering of NOS1 and NOS2 proteins in *D. pulex* and *D. magna* (Fig. 2) indicates that a NOS gene duplication predates the speciation of the two *Daphnia* species, which is thought to have occurred anywhere from 10mya [44] to 200mya [45]. *Daphnia* NOS genes are also strikingly divergent from other invertebrates, with Dmag-NOS1 showing amino-acid sequence similarity to other arthropods ranging from 63% to 67% (Figs. 2 and 3). As is the case for *D. pulex* [12], Dmag-NOS2 is even more divergent, with similarity to other arthropods from 55% to 59% (Figs. 2 and 3). The results from PAML analysis confirmed rate heterogeneity within the invertebrate NOS phylogeny (*χ*^2^ = 318, d.f. = 1, *p* ≪ 0.0001). As gene duplication may permit different rates of evolution, we tested whether or not this was the case in the *Daphnia* NOS genes. We found that the best model (*χ*^2^ = 76, d.f. = 1, *p* ≪ 0.0001) allowed for three different rates to be applied to the tree: one for all branches leading to *Daphnia* NOS1, a second for *Daphnia* NOS2 and the third for all other branches within the tree. The relative rate for these different groups was 1 (all branches, except those of the *Daphnia* NOS clade): 2.2 (*Daphnia* NOS1): 4.8 (*Daphnia* NOS2), leading us to conclude that the *Daphnia* NOS2 gene in particular is diverging faster than other invertebrate NOS genes.

The different rates of evolution between the *Daphnia* NOS genes may be a result of strong positive selection, which has been frequently observed in immunity genes [46–49]. We tested if this was the case by comparing divergence at synonymous sites (which should evolve essentially neutrally) to divergence at non-synonymous sites. However, as has been previously reported, the synonymous substitution rate (Ds) between *D. magna* and *D. pulex* was high [44]. Indeed, Ds between *D. magna* and *D. pulex* and *D. magna* and *D. parvula* indicated that the sequences were saturated for both of the NOS genes (*D. magna*/*D. pulex*: NOS1 Ds = 0.62, NOS2 Ds = 1.17, *D. magna*/*D. parvula*: NOS1 Ds = 1.03, NOS2 Ds = 0.55, all Jukes-Cantor corrected), precluding investigations into the type of selection acting in this species pair. By contrast, Ds values between *D. pulex* and *D. parvula* were suitable to evaluate Dn/Ds, and this comparison indicated that both genes are under purifying selection (NOS1 Dn/Ds = 0.14, NOS2 Dn/Ds = 0.10). However, as only a portion of each gene was sequenced, these results may not be representative of the selection pressure over the entirety of the gene sequence. Nonetheless, there is no indication that these genes are undergoing positive selection, as would be expected if one or both gene copies were involved in a coevolutionary arms race with a pathogen.

**Fig. 4:**
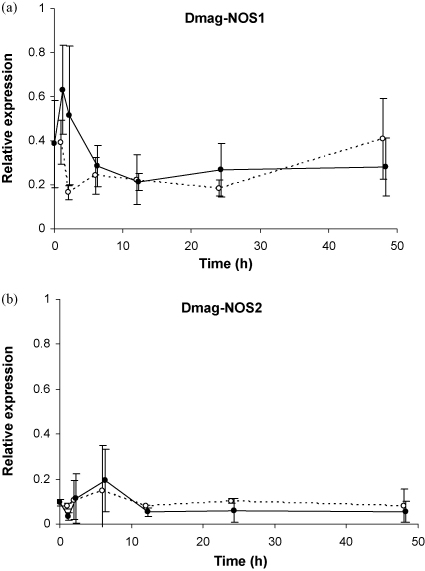
Relative Dmag-NOS1 and Dmag-NOS2 expression following pathogen exposure in the GG4 *D. magna* clone. Expression of (A) Dmag-NOS1 and (B) Dmag-NOS2 relative to *actin* is presented for exposed (full black circles, solid line) and not-exposed (white circles, dotted line) treatments. Each point corresponds to the mean of three independent replicates, the error bars shown are standard error. The black circles have been slightly shifted horizontally for easier reading. No statistically significant effect of pathogen exposure on the expression of either Dmag-NOS1 or Dmag-NOS2 was found, although a non-significant increase in Dmag-NOS1 was visible after 1–2 h (Exposure:Time*F* = 2.50, *P* = 0.12; Exposure*F* = 1.82, *P* = 0.19.

To assess intraspecific variation, we sequenced the complete cDNAs of both NOS genes from 14 different *D. magna* clones. For Dmag-NOS1, the standard protein is 1182 amino-acids long, but two clones (GG8 and GG15) each presented two transcripts, one encoding the standard protein and one containing a deletion of 92 nucleotides. Due to this deletion no viable protein can be translated from the usual start codon (frame shift). Alternatively, the short transcript might encode a 878 amino-acid-long protein, with an alternative start at the 306th amino-acid in the standard protein. Of 33 variable amino-acid sites within the 3546 bp Dmag-NOS1 ORF, nine contain non-synonymous mutations, among which 4 do not change the polarity of the amino-acid (Table 2A). No mutation appears to be associated with resistance as observed by Carius et al. [27] for the four Gaazerfeld german clones (“GG” clones) or with the patterns observed in the four Scottish clones (“K” clones, T. Little, unp. data). For Dmag-NOS2, possibly due to the low expression of this gene, we obtained the full sequence for only 11 of the 14 clones. However, the sequences obtained were sufficient to gain at least some insight into the variability of Dmag-NOS2. In the 3249 bp of the ORF (1083 amino-acids), we found 65 variable sites (Table 2B), which is greater than that observed for Dmag-NOS1 (*χ*^2^ = 12.10, *p* < 0.001). Of these, 26 were non-synonymous mutations, among which 16 do not alter polarity (Table 2B). Again, no mutation in Dmag-NOS2 appears to explain the resistance variability observed among the German or Scottish clones. Overall, both NOS genes were found at relatively low constitutive levels in our *Daphnia* extracts: Dmag-NOS1 and Dmag-NOS2 expression levels are about half and 10% of *actin* expression, respectively (Fig. 4A and B, time 0). This is not surprising considering that NO is potentially toxic [1,2,4,5].

**Fig. 5:**
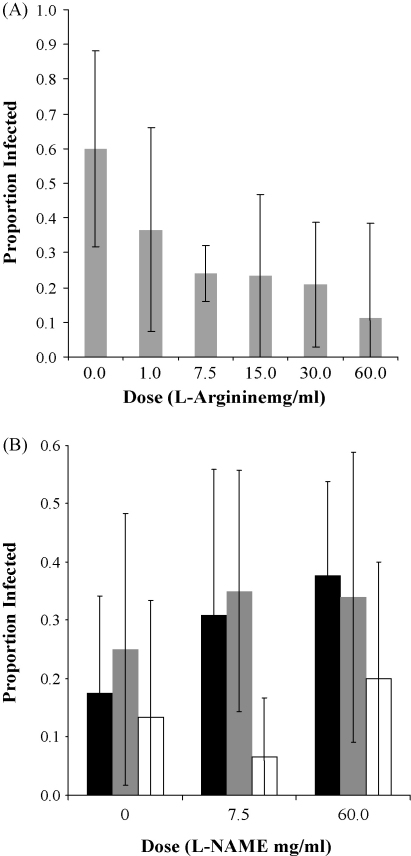
(A) Effect of increasing doses of l-arginine, the NOS substrate, on *P. ramosa* infection rate. For each dose of l-arginine (in mg/ml) added to the diet, the mean proportion of infected individuals is indicated; the error bars represent the standard error. Increasing the quantity of l-arginine had a significant effect in decreasing the number of *Daphnia* infected (*F* = 3.27, *P* = 0.006). (B) Effect of l-NAME, a NOS inhibitor, on *P. ramosa* infection rate. For each dose of l-NAME (in mg/ml) added to the diet, the mean proportion of infected individuals for each clone (GG4 in black, GG3 in grey and GG15 in white) is indicated; the error bars represent the standard error. There was no significant interaction between genotype and l-NAME dose (Clone:Dose, *F* = 0.87, *P* = 0.48), but there was a significant effect of the genotype (Clone, *F* = 7.29, *P* < 0.001). There was also a close to significant trend in an increase of infection rate when l-NAME doses increase (Dose, *F* = 2.44, *P* = 0.088).

To gain a first indication if NOS and NO could be involved in resistance to pathogens in *D. magna*, we performed two infection experiments. In the first, we analysed expression of both NOS genes in *D. magna* individuals exposed to the pathogenic bacterium *P. ramosa*. Although these infections were successful [26], we found no statistically significant effect of exposure on the expression levels of either Dmag-NOS1 or Dmag-NOS2 (Fig. 4). This has been confirmed in recent replicates of this experiment (data not shown). Second, we simultaneously exposed hosts to *P. ramosa* and to a range of concentrations of l-arginine, the substrate of NOS and the limiting factor of its activity, and to two concentrations of l-NAME, which inhibits the NOS pathway. It has been shown in the mosquito *A. stephensi* and in the crayfish *Procambrus clarkia* that resistance to pathogens increased when host diet was supplemented in l-arginine [11,20], and we found a similar pattern in *D. magna*: the proportion of individuals infected with *P. ramosa* decreases with the quantity of l-arginine available (Fig. 5A), but increases with the addition of l-NAME, although this increase in only close to significance and appears to be relatively clone-dependent (Fig. 5B: GG15 does not exhibit the same trend as the other clones, which could be linked to the fact that this clone is generally more resistant to *P. ramosa* [27]). This observation is compatible with the hypothesis that at least one of the NOS genes functions to fight infection in *D. magna*, although our expression experiment indicates that this is unlikely to be attributable to increased expression levels. However, levels of NOS proteins may be different and could be examined in a further study. Alternatively the NOS RNA could be stable enough so that the translation is sufficient throughout the infection period.

Other studies have indicated that NOS expression is increased in the presence of pathogens (or LPS which mimics pathogen presence, e.g. [8,11,16,17,19,20]). The possible role of NO in the invertebrate immune response to pathogens is reinforced by evidence of pathogen adaptation to host NO production: some pathogens appear to actively move to tissues rich in NO-scavenging proteins, while others induce the enzyme arginase, which competes with NOS for l-arginine substrate, thus inhibiting the host NO production [2].

In conclusion, we showed that two NOS genes are present in *D. magna* as the result of a relatively ancient gene duplication. The two NOS proteins differ in terms of expression level, polymorphism and divergence from the other arthropods. This could be explained by the specialization of the two proteins to different functions; NOS is implied in several disparate processes in vertebrates (see review in [1]), and this could be the case in invertebrates as well. If so, perhaps this ancient duplication is an example of neo- or subfunctionalization [50–52]. Ohno [50] first proposed that duplications provide new genetic material for evolution, as one copy may experience relaxed selective pressure. Often, new mutations will inactivate the new copy (leading to the creation of a pseudogene), but on rare occasions it could acquire a new function, i.e. neofunctionalization. Another model of evolution by duplication proposes that if a gene performs several functions (pleiotropy), a duplication may allow each daughter copy to specialize in a different subset of the original functions (subfunctionalization [53,54]; other models of evolution of duplication have been proposed, see for a review [55]). The rapid evolution of Dmag-NOS2 (see above and Fig. 2) led us to investigate whether this gene is subject to a host–pathogen arms race; however, the Dn/Ds values we observed do not support this. Further arguing against an arms race is the observation that Dmag-NOS2 has higher intraspecific variability than Dmag-NOS1 (arm-races cause selective sweeps, which diminishes polymorphism). Thus, NOS2 appears to experience relaxed selective pressure, but may be either acquiring new function or specializing on a subset of functions, potentially unrelated to immunity, while NOS1 assures that essential functions are still assumed. Whatever their mode of evolution, either or both of the NOS genes may still play a role in the immune response as indicated by our infection experiments and NO may yet have a role in resistance to *P. ramosa*.

## Figures and Tables

**Table 1:** Specific primers used to sequence each NOS full-length cDNA for the various *D. magna* clones.

Gene	Forward primer	Sequence (5′–3′)	Reverse primer	Sequence (5′–3′)
NOS1	SeqNOS1_F1b	GGCAGACCGAGCACTAGTCAGT	SeqNOS1_R1bc	GACGTGCGTCAAACACCTGTAG
	SeqNOS1_F2	ATCCTGAAGATGAGGAAGCCCTAC	SeqNOS1_R2	AAGACCGAGCTCAGAGAACCAAT
	SeqNOS1_F3	TCAGCTACGCAGGTTATCGAAATA	SeqNOS1_R3	GAAAAACGGGCGTCAGAGAAC
	SeqNOS1_F4b	CGCATACTAGCCTCTGGAAGGA	SeqNOS1_R4bc	ATGGTGGTGTGAGTGGTGATTG
	SeqNOS1_F5	TCAAGTATCGCTTCAAAGAGGTTG	SeqNOS1_R5	CAGGACTTGAAGGTTTGCTCTTG
	SeqNOS1_F6	CGCTACTTAACCGAGTTCTCAACC	SeqNOS1_R6	AATCAGTCCGGTCACTAGGGTATC
	SeqNOS1_F7	CAAGAGAAGCCGATTCACGATATT	SeqNOS1_R7	GAAATGCTGTAGAACCTAGGCTGA
	SeqNOS1_F8	GTCACAGACCCTCTTGGCTCTACT	SeqNOS1_R8	TCTTGTACGTAAGTCTTGGGAACG
	SeqNOS1_F9	TCACTTACCTACCGAAATTGAGAGG	SeqNOS1_R9	ATAAAAATGAAAAGTCCGCCAAAG

NOS2	SeqNOS2_F1	CGTGGATCATCATTCGATTC	NOS2_R2-1	CCAGACACGAAAATCGGACT
	SeqNOS2_F1b	TTCTGGGTTTCCAACTTTTTGC		
	SeqNOS2_F2	TACGATGAGCTGGTGTACGG	NOS2_R4-2	GAGCACCGCTTTATTCACCT
	SeqNOS2_F2b	CGAACACATCAATTACGCAACA		
	NOS2_F2-1	AGTCCGATTTTCGTGTCTGG		
	NOS2_F3-2	GCGGTAGCCAATATGCTGTT	SeqNOS2_R3	TTTTGAATTCATCCCCGTTG
	NOS2_F5-2	CCAATACCCCCAAAAAGTTG	NOS2_7R-2	CTTGGTTGTTCCGAGGAAAG
	NOS2_F6-1	CGGGGATGAATTCAAAAAGA	NOS2_R8-3	CGACGGATTTCTTCCAGGTA
	NOS2_7F-2	CTTTCCTCGGAACAACCAAG	NOS2_R9-5	CCGTCTTTCCAGAGCAAGTC
	SeqNOS2_F7c	GGAGATTTCGTAGCGTGCTATTTC	SeqNOS2_R7b	TAGGCCTACTAGCCTTGGTGGT

**Table 2:** cDNA sequence variation between *D. magna* clones. Each variable site is indicated with its position relative to the first nucleotide of the first methionine, (A) for NOS1 and (B) for NOS2. GG3 cDNA is used as the reference sequence, a dash (-) indicates no differences with the reference. Nucleotides R, K, M, W, S and Y correspond to heterozygote sites (IUB ambiguity codes). For non-synonymous mutations, the corresponding amino-acid changes are indicated. For NOS1, the deletion (Del.), observed for some transcripts (see text), is boxed, and nucleotide changes before that deletion are lower cased. For NOS2, a dot (.) indicates that the sequence is missing for that clone.

Clone	Position																																			
																1	1	1	1	1	1	1	1	2	2	2	2	2	2	2	2	3	3	3	3	3
		1	1	2	2	2	2	4		5	6	7	8	9	9	2	2	3	3	3	6	9	9	1	1	1	3	5	6	8	8	1	2	2	4	5
	4	6	8	3	3	5	7	8	-	7	7	9	7	5	9	3	5	0	0	1	5	3	8	1	5	6	7	2	7	0	6	9	2	7	3	4
	9	5	0	1	9	7	6	0		1	0	4	9	4	1	3	1	5	8	4	3	2	9	2	4	3	0	9	6	5	2	3	6	4	0	0
(A) NOS1																																				
GG3	G	T	Y	K	T	A	M	GxxT			G	S	Y	W	A	R	T	M	C	W	G	G	C	T	C	C	C	R	A	G	G	G	C	A	T	G
GG4	-	-	T	G	K	-	C	-			-	-	-	-	-	-	-	-	-	T	-	-	-	-	-	-	-	-	-	-	-	-	-	-	-	-
GG7	-	-	T	G	-	-	C	-			-	-	-	-	-	-	-	C	-	-	-	-	-	-	-	-	-	-	-	-	-	-	-	-	-	-
GG8-long	C	-	T	T	-	-	C	-			-	C	C	A	-	G	-	A	-	T	-	-	-	-	-	-	-	-	-	-	-	-	-	-	-	-
GG8-short	c	-	t	t	-	-	c	Del.			-	C	C	A	-	G	-	A	-	T	-	-	-	-	-	-	-	-	-	-	-	-	-	-	-	-
GG13	-	-	T	G	-	-	C	-			-	-	-	-	-	-	-	-	-	-	-	-	-	-	-	-	-	-	-	-	-	-	-	-	-	-
GG15-short	c	-	t	t	-	-	c	Del.			-	C	C	A	-	G	-	A	-	T	-	-	-	-	-	-	-	-	-	-	-	-	-	-	-	-
GG15-long	-	-	T	G	-	-	C	-			-	C	C	T	-	A	-	C	-	A	-	-	-	-	-	-	-	-	-	-	-	-	-	-	-	-
KA5	-	-	C	G	-	-	A	-			R	-	T	-	-	G	A	A	-	T	-	-	-	-	Y	-	-	A	R	R	R	-	-	G	C	A
KA51	-	-	C	G	-	-	A	-			-	G	T	T	-	G	A	A	-	T	-	-	-	-	-	-	-	A	G	A	A	-	-	G	C	A
K24	-	Y	C	G	-	-	A	-			R	-	T	-	-	G	A	A	-	T	-	-	-	K	Y	Y	Y	A	R	R	A	R	Y	R	Y	-
K47	-	-	C	G	-	-	A	-			R	-	T	-	-	G	A	A	-	T	-	-	-	K	Y	Y	Y	A	R	R	R	R	-	R	Y	R
BelgiumD1	-	-	C	G	-	R	C	-			-	-	T	T	-	-	-	-	-	-	-	A	Y	K	Y	Y	Y	A	R	-	-	R	Y	-	-	-
BelgiumD3	-	-	T	G	-	-	C	-			-	C	C	T	-	-	-	-	-	-	R	-	-	-	-	-	-	-	R	-	-	-	Y	-	-	-
Munich	-	-	C	G	-	-	A	-			-	G	T	T	-	G	A	A	-	T	-	-	-	-	-	-	-	A	G	A	A	-	-	G	C	A
Finnish	-	-	C	G	-	-	C	-			-	C	C	A	T	G	-	C	T	T	-	A	-	-	-	-	-	A	G	-	-	-	-	-	-	-

	V				L	E					N	P			T																	E		T	S	
Amino-acid change	↓				↓	↓					↓	↓			↓																	↓		↓	↓	
	L				R	G					D	R			S																	K		A	P	

Clone	Position																																			
																		1	1	1	1	1	1	1	1	1	1	1	1	1	1	1	1	1	1	1
						2	2	2	3	3	3	3	4	7	8	9	9	0	1	1	1	1	1	1	1	2	2	3	4	5	5	6	7	8	8	8
	1	3	4	7	9	2	4	5	6	6	6	7	2	0	5	7	9	0	1	3	3	5	7	8	9	4	6	0	4	2	5	1	2	0	0	0
	3	3	8	8	6	3	3	8	0	6	9	8	0	9	8	8	3	5	3	4	7	0	3	8	4	8	3	5	4	1	4	7	1	0	2	3
(B) NOS2																																				
GG3	G	A	A	C	G	A	C	T	G	T	C	G	G	C	A	G	C	T	A	G	C	T	G	G	A	T	T	A	T	C	C	T	G	G	C	C
GG4	-	-	T	T	-	-	-	-	-	-	G	-	-	T	R	-	-	-	-	A	-	-	-	A	-	G	-	-	-	-	-	C	-	-	-	-
GG7	-	-	T	T	-	-	-	-	-	-	G	-	-	T	G	-	-	-	-	R	-	-	-	R	-	K	-	-	-	-	-	C	-	-	-	-
GG8	-	-	W	Y	-	-	Y	-	-	-	S	-	-	T	G	-	-	-	-	A	-	-	-	A	-	G	-	-	-	-	-	C	-	-	-	-
GG13	.	.	.	.	.	.	.	.	.	.	.	.	.	T	G	-	-	-	-	A	-	-	-	A	-	G	-	-	-	-	-	C	-	-	-	-
GG15	-	-	T	T	-	-	-	-	-	-	G	-	-	T	G	-	-	-	-	A	-	-	-	A	-	G	-	-	-	-	-	C	-	-	-	-
KA5	-	G	-	-	-	-	-	C	-	C	-	A	-	T	-	-	A	C	-	A	-	-	-	-	-	G	-	-	G	-	-	C	T	A	-	T
KA51	-	G	-	-	-	-	-	C	-	C	-	A	-	T	-	-	A	C	-	A	-	-	-	-	-	G	-	-	G	-	-	C	T	A	-	T
K24	-	G	-	-	-	-	-	C	-	C	-	A	-	T	-	-	A	C	-	A	-	-	-	-	-	G	Y	-	G	-	-	C	T	A	-	T
K47	-	G	-	-	-	-	-	C	-	C	-	A	-	T	-	-	A	C	-	A	-	-	-	-	-	G	-	-	K	-	-	Y	K	R	-	Y
BelgiumD1	R	R	-	-	R	M	-	Y	-	Y	S	R	K	T	R	-	-	Y	-	A	-	-	-	-	-	K	-	-	-	-	Y	Y	T	R	S	-
BelgiumD3	-	-	-	-	-	-	-	-	-	-	S	-	-	T	G	K	M	Y	T	-	T	-	-	-	-	-	-	-	-	-	-	Y	-	-	-	-
Munich	-	G	-	-	-	-	-	C	-	C	-	A	-	T	-	-	A	C	-	A	-	-	-	-	-	G	-	-	G	-	-	C	T	A	-	T
Finnish	-	G	-	-	-	-	-	C	A	C	-	A	-	T	-	-	M	Y	-	-	-	Y	K	-	W	-	-	R	K	Y	-	C	T	-	-	-

	D					H							Q						E			N	M						F				R		T	
Amino-acid change	↓					↓							↓						↓			↓	↓						↓				↓		↓	
	N					N							H						D			R	I						V				I		S	

Clone	Position																												
	1	1	1	2	2	2	2	2	2	2	2	2	2	2	2	2	2	2	2	2	2	2	2	2	2	3	3	3	3
	8	9	9	0	0	1	1	1	2	2	4	5	5	5	5	5	6	6	7	7	7	8	8	9	9	0	1	2	2
	2	2	2	7	9	0	2	4	4	9	5	0	4	4	8	8	5	8	5	5	9	4	6	4	6	8	0	0	4
	6	8	9	3	8	9	1	7	6	9	5	3	1	5	5	8	5	9	0	1	5	7	5	4	8	7	5	1	4
(B) NOS2																													
GG3	C	T	C	A	A	T	C	T	A	A	T	T	C	G	G	T	T	T	G	G	A	G	T	G	A	T	A	G	G
GG4	-	-	-	C	-	C	-	.	.	.	.	.	.	.	.	.	.	.	.	.	.	T	C	-	G	A	-	A	-
GG7	-	-	-	C	-	C	T	-	C	G	C	C	-	-	A	G	C	C	-	-	T	T	C	-	G	A	-	A	-
GG8	-	-	-	C	-	C	T	-	C	G	C	C	-	-	A	G	.	.	.	.	.	.	.	.	.	.	.	.	.
GG13	-	-	-	C	-	C	T	-	C	G	C	C	-	-	A	G	-	C	-	-	T	K	C	-	G	.	.	.	.
GG15	-	-	-	C	-	C	T	-	C	G	C	C	-	-	A	G	C	C	-	-	T	T	C	-	G	A	-	A	-
KA5	A	-	-	-	-	-	-	-	-	G	-	-	-	-	-	-	-	-	-	A	-	-	-	A	-	-	-	-	-
KA51	A	-	-	-	-	-	-	-	-	G	-	-	-	-	-	-	-	-	-	A	-	-	-	A	-	-	-	-	-
K24	A	-	-	-	-	-	-	-	-	G	-	-	-	-	-	-	-	-	-	A	-	-	-	A	-	-	-	-	-
K47	M	-	-	-	-	-	-	-	-	G	-	-	-	-	-	-	-	-	-	A	-	-	-	A	-	-	-	-	-
BelgiumD1	M	-	-	M	R	Y	-	-	-	-	Y	Y	M	R	R	K	C	C	-	-	-	K	C	-	R	W	W	A	S
BelgiumD3	-	-	-	-	-	Y	-	-	-	R	Y	-	-	-	R	K	Y	Y	S	-	-	-	Y	-	R	-	-	-	-
Munich	A	-	-	-	-	-	-	-	-	G	-	-	-	-	-	-	-	-	-	A	-	-	-	A	-	-	-	-	-
Finnish	-	A	G	-	-	-	-	C	-	G	-	-	-	-	-	-	C	-	-	-	-	-	-	A	-	-	-	-	-

	A	I		K			I	K	G	S			V	C	L		S	G		K	W		V	V				A	
Amino-acid change	↓	↓		↓			↓	↓	↓	↓			↓	↓	↓		↓	↓		↓	↓		↓	↓				↓	
	E	K		E			T	T	S	P			I	Y	R		P	A		M	C		M	I				P	
