# Supplementary material for: An ancient immunity gene duplication in Daphnia magna: RNA expression and sequence analysis of two nitric oxide synthase genes
Source: Dev Comp Immunol. 2009 Sep;33(9-3):1000–10. doi: 10.1016/j.dci.2009.04.006 (PMC2724039; doi:10.1016/j.dci.2009.04.006)
Supplement: Supplementary file 1 [file mmc1.doc]

**An ancient immunity gene duplication in *Daphnia magna:* RNA expression and sequence analysis of two Nitric Oxide Synthase genes**

**Pierrick Labbé, Seanna J. McTaggart and Tom J. Little**

**Supplementary material**

**S1: Models for NOS genes in *D. pulex* refined using *D. magna* sequences.**

**I- *D. pulex* NOS1, refined gene model (February 2009)**

scaffold 20

join(1068965..1069045,1069129..1069320,1069397..1069545,1069621..1069736,1069810..1069897,1069983..1070193,1070267..1070436,1071042..1071341,1071454..1071598,1072021..1072195,1072471..1072598,1072704..1072787,1073470..1073574,1073823..1073871,1074033..1074256,1074410..1074551,1074950..1075002,1075092..1075193,1075313..1075487,1075698..1075840,1075922..1075983,1076108..1076190,1076476..1076567,1077521..1077683,1079838..1079998,1081698..1081853)

1 atgacttcca aactaaacgg cggcttggaa tcggaagatg gacatcagca ggtgcaaatg

61 catccaccgc cgccgtcacg caacaaacca atccgcctaa aaaatcattc cgctgtcatc

121 gagtcgtacg acagtctcta ccgcacagct aatccggaga gcggatgcgg atcgcaagtt

181 tgtcgggcga gtatctggca cttgacggac ccgcgtctgt cttatccaac accccgagag

241 ctactacccg aagatgaaga agcccttctc acttccgcca aagaatttct cgaacattat

301 tattcatcta ttaaaaggtt ggacacggaa tcacatcgag cccgatggga atctgtacgt

361 cgggacgttc acctgaccgg cacctacgac ctgactgaga cggaattgac gttcggcgca

421 aaacttgcct ggaggaattc agcccgctgt atcggccgca tccagtgggc caaactacaa

481 gtgtttgacg cacgtcacgt caccacggca cagggcatgt ttgaagctct ttgtaaccat

541 atcaaatacg ggacaaacaa aggcaacatt cgttctgctg cgaccatctt ctgccagcga

601 aaaccgggcc agggtgatta ccgcatttgg aacgcccaat tgatcagtta cgcaggttat

661 cggaatatgg aagatggcac cgtcatagga gatccggcga cggtggaatt caccgaagtg

721 tgtcaaaaat tgggatgggt cggagcggga acgcgattcg acttgctgcc cttagtcatt

781 caagctagta accgtctcta tccggaagtc ttcacccttc cgccagaact cgtcatggaa

841 gtgccactcc atcacccgac gtacgattgg ttcgccgagc tcgggcttcg ctggtatgcc

901 cttccagctg tgtccagcat ggcctttgat tgcggtggac tccagtttac ggccgtgccc

961 ttcaacggct ggtacatgac caccgagatc gccactcgcg atctgggcga cacgcatcga

1021 tataatcaac ttgagattgt ggctaagcga atgggcctgg acactcggac gcactccagt

1081 ctgtggaagg atcgggccgt catcgaaatc aacgccgccg ttctctacag ctatcagaaa

1141 atgaacgtga ctatcgtcga tcatcacaca gcctccgaat cttttatgaa acatatggaa

1201 acagagtttc gactaagagg cggctgtcca gccgagtggg tctggatcgt cccgcccacc

1261 tccggttctc tgacgcccgt ttttcatcag gaaatggttt gctattccct caagccaagc

1321 ttcgaatacc aggaggtggc ttggaagaat ttccagtggg atgaagaaga cacgaatacc

1381 ggctctgcgt tggggttgct ggccgtcgga cgaagtgcgg gaggacgtcg tgtcaagtat

1441 cgtttcaagg aggttgcacg ggccgtcaag ttcacttcga atcttttcgg caaagccctg

1501 caacgtcgta tcaaagccgc catcctctac gccaccgaga cgggcaaatc agaaaagtac

1561 gctcacatgc tggccgagct cttcaaccac gctttcaatg cacaagtcat gtgcatggcg

1621 gattacgacg tcatcaatct tgagcacgaa gcgctggtga tgatcgtgac gtcgacgttc

1681 ggcaacggag atccaccgga gaatggagag gcgtttgccc gtgaagtgcg cggaatggcc

1741 aacaaccacc actcacatca tcccatagct actcaaccta aatatcaact cgggtcttcc

1801 aagaccggca ctccgcttct taatcgagtt ctcaaccgcg atcagcatag cttgcgcagt

1861 ttttcgttcg atcaactcca acagccctcg gtcgacagtc tcggacctct aagtaatgtc

1921 aggtttgccg tgtttgctct gggatccagc gcgtatccaa atttctgtgg tttcggcctc

1981 tacatcgacg atctactagg aagcctcggc ggcgagaggc ttgcctcagt catttgcggc

2041 gacgaactga gcggccagga gcaaactttc aaatcatggg cccagcaagt cttccacacg

2101 gcttgcgaga ctttttgttt ggaagacgac gtcaatatag acgacgtcag tgccacattg

2161 cgcagagatc ttattacttc ggacacggtt cgcttcgcta tcgtcaactc ccaggagaaa

2221 ccaatcctcg atattgttgc aggcttgtcg cagacgcacg ccaagaacgt caccgaatgc

2281 ccggtcgtct ttaacgtgac gttagcaaag gacattgatg gtcgtaaaac tgttcacgtt

2341 ggactgaaaa tcaccagcca gtggatcaac ggaactcgca actcggcgac gtcggcggtc

2401 acctacgagc cgggtgatca cattggcatt tttgctgtca acgacgaggc cctagtgact

2461 ggactgattg atcgacttgg catcaattct accctgccac ccgacggccc tctccagctg

2521 caaacacttg cagagcagaa agatggtggg aaaacgtggc agaatcaaga acgcctgcca

2581 gcttgcagtt ttcggacgct cttgtcccat tatctggata tcacaactcc ggcgtcgcag

2641 acccttctgg ccctgcttgc agctcacgcc tcttccgagg aagacaagcg acgcctccag

2701 cttcttgcaa ccgacgctag tgaatacgaa gattggaaac actggggctt tcctcatctg

2761 ctggaaactc ttttagaatt cccttcagtt gccatagacg ctgctctact cgtgtctcaa

2821 cttcctctac ttcaacctcg tttctacagt atctcctcat ctccgctgat cgatccactc

2881 caaatcgata ttaccgcagc cgtcatcgcc ttcaaaacgc aaggtggcga aggtcccatg

2941 cactacggcg tttgctccag ttacctttct caggtgaacg aaggcgataa aatccagtgc

3001 ttctttcgca gcgcacccaa ttttcactta ccaaccgaaa ttgaaagacc agtggttatg

3061 gtcggtccgg gtaccggtgt ggctcccttc cgtggctttt ggcaacacaa acaagccctt

3121 caatcgaaag ggaaagagat ggggcccatg ctgttgttta cgggctaccg ttctccggat

3181 tgcgatctgt tcgtcgaaga aaaatcgacc atggttgcct cgggaatttt agactacgcc

3241 tttttggctc tatcccgcca cccacccgtt cgcaagactt atgtccaaga caagttgctg

3301 gaagccgctc cgctagttta tcgaatgttg actcagcaaa tgggtcattt ttatgtgtgt

3361 ggcgactgcg cgatggccga agacgtggcc aacactttgc gaatggtgtt tcagaaagcc

3421 ggcggtttga atgccgaaga atcggatgac tttctcatgc aactgcgaaa cgagaggcgt

3481 tatcaggagg acatttttgg tatcacttat cgggcgcccg acacggtcaa tgccaacaga

3541 cgtcagcaa

**II- *D. pulex* NOS2, refined gene model (February 2009)**

scaffold 11

join(565951..566040,566163..566305,566397..566559,566686..566934,567041..567183,567265..567427,567885..568049,568126..568304,568382..568561,568922..569276,569354..569529,569594..569797,569875..569978,570066..570214,570293..570484,570559..570600)

Assembled scaffold has a gap from 567438-567875. Primers designed in exons on either side of this gap were designed and amplified in several *Daphnia pulex* individuals. This resulted in an additional exon being discovered. The following sequence includes this extra exon, which is not found in the *Daphnia pulex* genome assembly. This exon is written in CAPITAL letters, and includes ambiguity codes for polymorphic sites.

1 atgtggttgg aagatcgacg cggcgttgaa ctacgcaatc acacaacagg tgaagtttcc

61 gttgacaagc tgtttcatga aactatttgt gaaggacctt gttcgggaga agaagtttgc

121 gtcggcagtc gaatggtccc cagacaagtc cccgccaatt acaaacgacc aaaagacaaa

181 attttgcaac aggccaagga gtttctcgat ggatacttcc gtgcgtcaaa aaaatttgct

241 tcagaggaac atattgtgcg gttacttcaa gttcacaagg acattgagaa tcatggtaag

301 tatgatttga cttacgatga gttagtacac ggatgtggca tggcttggcg caatgcccca

361 cgatgcgtcg caagagttca gtggcaatcc ctcaaggtct tcgatgctcg gcatgttcaa

421 acggcccaag aaatgttcga tagccttgtc gagcacatca agtacgcaac aaatcaaggg

481 cgtcttcgtt cagtcataac cgtctttcca ccccgcactg acggccagct cgactttcgt

541 atctggaaca gccaattgat tagttacgct ggctatcgga acccggatgg aacaatacaa

601 ggagatcctt ccaacgtcca gtttactgag gcatgtttta tatatctgtg tctctcattg

661 ggatgggata gacaagagag aactcaattc gatattctcc cccttgtcct tcaagctaag

721 ggtcttgcgc cccaagtttt cttacttccg gatgacattg tacttcaagt tcctctcgtg

781 catccatcgt tgccttggtt cggagaattg ggattgcgtt ggtacgccct acctgcggta

841 gccaatatgc tctttgactg tgggggtatc caatttccag ctgctccctt taacggatgg

901 tatatgagca cagagatagg ctctagagat ctgtgcgatc ctcaacgcta tAATATATCA

961 GAAATGATAG CCAGAAAAAT GGAATTrGAC ACGAGCACTC ACGCCACACT TTGGAAAGAC

1021 ACAGTTTTGy TGGAGGTGAA CAAAGCGGTG CTGTACAGTT ATCAAAAGTG TGGGGTGACT

1081 ACCTGGGACC ATCATACGGC CTCTGAATCT TTCATCCAGT TTTACGCAAA TGAAATGAAG

1141 ACGCGCGGAG GATGCCCAGC CGATTGGATT TGGCTTGTCC CGCCAATGTC TTCTTCTTCT

1201 TCGCCACTTT TTCATCAAGA AATGGCCTTT TACTTTCTCA GGCCGTCTTT CGAATACCAA

1261 ATGGCAGCCT GGTCAAATTT CCAGACGAAA TCATTAGTCA AAAGTAGAGG ACAAGGTAAC

1321 CCGAAGAAGa aaatgatggc agtaattata gcggttcgac tgtgcagtta tttgttctcc

1381 tctcgttggt cgaagcgacc caaagtggtc gtcctatttg cttcacaatc cggaagagct

1441 gaaaactttg cgacgaaagc attcgtcaag ttcaaatctc ttttccaggc caatctttac

1501 tgtatggctg attttgatac caagaaattg caagatcaac aggcaatcat tgtagttgca

1561 tcgacgttcg gagacggaga ggcccccaac aatggagaag aattcaaaac aaatctgatt

1621 aaaatgcaca aggaaaactt taaatttcga cacatcaaca atcaactccc atgttttgcc

1681 gttctgggtc tgggatcgac atcatatcac caattttgcg cgttcgggaa atttgtcgac

1741 gtaacgttta aagaactggg gggaacccgt ctgttgccgc tcgcctgtgc cgacgaactt

1801 aacaatcaag aaaaaactgt tcagacttgg ttgtcagaca taactactgc gctccgagac

1861 ggtcatgcaa caatcctcct tcagttaaac acattaaacc cggaaacact caagtacaaa

1921 cccggagatc acttggccgt cttccctcgg aataatcgag acatgatgca acgctttgta

1981 cttcgtctaa gaaactgtcc cgcgtccaaa tccgcaacga ttcaacttca agttcaggga

2041 cccaatggaa acagggaaaa ttggaagaga attccgccgt gttcgtttga caatttactg

2101 gctcgattca tagatttgac cagcccacct tcacagacta ctcttcgact tatggcttcg

2161 acagcaatcg atcaaaaaga taaaatgcga cttgaaattt tagccgagga tttggcagct

2221 tatcaaaaat gggtatcatc cgttaatcct aatattgtgg acgtcttgga ggactttctt

2281 tccgtcgatg tcgatgcggc ggctttaatc agcacgctac caattattca acctcgtttc

2341 tacagtattt cctcttccgc tgtctttgca ccgactgaaa tccatctaac agcaagttta

2401 gttcgttacc ggactgaagc tgacaaaggc cgtcttttcc acggactttg tacttcttac

2461 ttggaacaaa ccaatcgagg agattttata atttgctact tcaagtccaa tccaagcttt

2521 catttaccac cctatccgtt aaagcccatc atttgtatcg ctgctggaag tggtatcgcc

2581 cctttaagag gtttctggca acaaagacat tttgaaaagg gcgcatttca tcacactttc

2641 atctccaaac tgtgggggaa tttttcgaaa cgacctgtcc agcagcacca atccaccaat

2701 cagtatggtg caatctacat gtactacgga tgcagagaaa aaacttcaca gccgtttcgt

2761 aatgaactgg atacgatgat gcaacataag gtcatcacta aaacctttgt ggcattttct

2821 agagaaaccg caaagccgaa agaatatgtt caagatttgc tttggaagga cggtgctcgg

2881 gtgtctactc aaattttaaa cgagggagct tacgtgtaca tttgtgggaa gacagcgatg

2941 gccacccaag tggaagagac tatcattcgg attattcgtc aatacggcga aatgaatcac

3001 gacgaagcgg aaatggtctt cagaaatctt aaagcggggg gtcgttacaa gactgacata

3061 tttggatcaa aatag
